# Implicit learning modulates attention capture: evidence from an item-specific proportion congruency manipulation

**DOI:** 10.3389/fpsyg.2014.00551

**Published:** 2014-06-04

**Authors:** David R. Thomson, Karen Willoughby, Bruce Milliken

**Affiliations:** ^1^Department of Psychology, Neuroscience, and Behavior, McMaster UniversityHamilton, ON, Canada; ^2^Department of Psychology, University of WaterlooWaterloo, ON, Canada

**Keywords:** attention capture, implicit learning, visual search, proportion congruency, episodic retrieval

## Abstract

A host of research has now shown that our explicit goals and intentions can, in large part, overcome the capture of visual attention by objects that differ from their surroundings in terms of size, shape, or color. Surprisingly however, there is little evidence for the role of implicit learning in mitigating capture effects despite the fact that such learning has been shown to strongly affect behavior in a host of other performance domains. Here, we employ a modified attention capture paradigm, based on the work of Theeuwes (1991, 1992), in which participants must search for an odd-shaped target amongst homogeneous distracters. On each trial, there is also a salient, but irrelevant odd-colored distracter. Across the experiments reported, we intermix two search contexts: for one set of distracters (e.g., squares) the shape singleton and color singleton coincide on a majority of trials (high proportion congruent condition), whereas for the other set of distracters (e.g., circles) the shape and color singletons are highly unlikely to coincide (low proportion congruent condition). Crucially, we find that observers learn to allow the capture of attention by the salient distracter to a greater extent in the high, compared to the low proportion congruent condition, albeit only when search is sufficiently difficult. Moreover, this effect of prior experience on search behavior occurs in the absence of awareness of our proportion manipulation. We argue that low-level properties of the search displays recruit representations of prior experience in a rapid, flexible, and implicit manner.

## Introduction

When passively gazing around a new environment, or examining a piece of artwork, our eye seems to be drawn first to the most “unique” aspects of the scene. That is, we might find ourselves fixated on a brightly colored building or an oddly shaped figure. Although it may feel as though our attention is being “grabbed” or “captured” by these salient percepts in an entirely automatic manner, a host of empirical work has now demonstrated that several factors influence the deployment of visual attention. Aside from stimulus salience, these include the explicit, conscious goals and intentions of the observer, as well as the implicit effects of prior experience and context (Awh et al., 2012). These so-called “top-down” factors have been the focus of recent empirical investigations as researchers attempt to understand the extent to which prior knowledge and experience guides visual attention (see Lamy and Kristjánsson, 2013, for a recent review). The present work seeks to determine whether learned context can affect the deployment of attention toward, or away from, salient objects in a *flexible* (i.e., trial-to-trial) manner, and if so, under what conditions.

Theeuwes (1991) first demonstrated the automatic deployment of attention toward salient objects in the context of visual search. When participants searched for an odd shaped item amongst homogeneous distracters, the presence of an irrelevant color singleton significantly slowed search times. That is, despite the fact that observers were attempting to ignore the irrelevant singleton, they could not help but attend to it. This effect persisted even with extended practice (Theeuwes, 1992), and with advanced cueing of the target dimension (color or shape; Theeuwes et al., 2006; Theeuwes and Van der Burg, 2011). These findings led to the conclusion that top-down knowledge, defined by explicit intentions and/or expectations, cannot override the capture of attention by the most salient item in a search display. Put differently, parallel search (that is, search for a feature singleton, see Treisman and Gelade, 1980) unfolds in an entirely “bottom-up” manner.

Bacon and Egeth (1994) challenged the notion that the guidance of attention by bottom-up salience is impenetrable to top-down influences. It was argued that because the target item in Theeuwes' experiments was itself a feature singleton, subjects adopted a search strategy in which they allowed attention to be guided by singletons in the display. As a result of this strategy (known as singleton search mode) they allowed attention to be drawn by the singleton distracter, which was the most salient feature singleton in the display. Bacon and Egeth tested this idea in two-ways. In one experiment, participants searched for a predefined target shape (e.g., Circle) presented amongst distracter shapes (e.g., Diamonds), but on most trials there was actually more than one target shape. Crucially, when more than one target shape was present in the display, the target shape was no longer a shape singleton, which would presumably discourage use of a singleton search strategy. Instead, participants might rely on search for a particular form (known as feature search mode). Indeed, with multiple identical shape targets, search times were not slowed by the presence of the irrelevant color singleton, consistent with the idea that “search mode” determines whether attention is captured by an irrelevant color singleton. In a following experiment, Bacon and Egeth (1994) discouraged singleton search mode by presenting multiple shapes as distractors, again with the idea that the target shape would no longer be a shape singleton. Here also, search times were not slowed by the presence of the irrelevant color singleton.

A similar set of findings was reported by Leber and Egeth (2006) in a task in which subjects were trained on trials in which only feature search was available as a search strategy and were then transferred to a test phase containing trials identical to those employed by Theeuwes (1991, 1992). This manipulation was intended to counter claims made by Theeuwes (2004) that when the target item is a singleton, attention will always be captured by an irrelevant singleton (provided that it is more salient than the target). Crucially, in Leber and Egeth's experiment, the capture of attention by the irrelevant singleton was again absent on the test trials, implying that participants learned to use feature search mode despite the fact that singleton search mode was available on the test trials.

Numerous other recent findings converge on the idea that attention capture by an irrelevant singleton is not obligatory, and is in fact subject to top-down influences. For example, Turatto and Galfano (2001; Experiment 2B) found that when participants were told that the location of an irrelevant color singleton was negatively correlated with the location of the target shape singleton, attention capture effects were overridden. In addition, Zehetleitner et al. (2012) have shown that extended practice with an invariant color singleton mitigates the observed capture effect. Similarly, Burra and Kerzel (2013) observed reduced behavioral capture and a reduced N2pc waveform (which is argued to index a response to “conflict,” which would occur during capture) when singleton targets were predictable, relative to when they were not. Finally, Geyer et al. (2008) found that capture effects varied depending on the proportion of trials within a block in which a salient distracter was present, with larger effects observed when singleton distracters were infrequent. Taken together, these studies converge with the studies on search mode (Bacon and Egeth, 1994; Leber and Egeth, 2006) to suggest that expectations and intentions *can* serve to modulate or override attention capture.

### Attention capture and prior experience

Another area of study in which evidence of control over attention capture has accrued focuses on implicit influences of prior experience on attention capture. Of course, the general idea that automatically retrieved prior experiences can guide cognition is not a new one. Logan (1988, 1990) argued that as experience with a task accrues, superficial aspects of the task (i.e., contextual factors) automatically recruit similar prior experiences from memory, and that these experiences (or “instances”) bias behavior toward actions that have been successful in the past (see also, Hommel, 1998, 2004). In general, the greater the contextual match between current and prior experience, the greater the probability of retrieval, and subsequent execution, of prior action.

In the domain of singleton search, there is ample evidence that a preceding identical singleton can speed response to a current singleton (i.e., “priming of pop-out” effects; Maljkovic and Nakayama, 1994). Some researchers have argued that this effect may owe, at least in part, to the automatic retrieval of similar prior episodes (Hillstrom, 2000; Huang et al., 2004; Thomson and Milliken, 2011). For example, it has been shown that the speed with which singleton search unfolds depends on whether the features of the target match those in the most recent *contextually similar* trial (Thomson and Milliken, 2012, 2013a). Additionally, when many prior experiences are contextually similar to the current one, priming effects are dependent on the number of intervening experiences that occurred between the current and “influencing” trial, which has been interpreted by some as evidence for a form of retrieval interference (Thomson and Milliken, 2013b; Experiment 3). It has also been shown that the benefits and costs of target feature repetitions and alternations vary depending on whether one is in a search context in which target repetitions are likely or unlikely (Geyer and Müller, 2009; Thomson et al., 2013).

Whereas an episodic retrieval interpretation of these trial-to-trial effects on visual search has been favored by some researchers, others have highlighted the possibility that such effects owe to more than one process (Ásgeirsson and Kristjánsson, 2011). For example, according to dual-stage or “hybrid” accounts (Lamy et al., 2010), attentional guidance early in the search process may be affected by prior “activation”/“suppression” of related low-level perceptual representations (see Lee et al., 2009), whereas attentional guidance later in the search process may be affected by the retrieval of episodic representations. In any case, the available literature on trial-to-trial influences on search provides compelling evidence that search behavior can be affected by context-dependent learning processes.

A study that offers converging evidence for this general idea, but that used a task more typical of those used to examine attention capture (e.g., Theeuwes, 1991, 1992) was reported recently by Cosman and Vecera (2012). Participants were exposed to two different contexts in a training session. In one context, they searched for a pre-defined shape target amongst heterogeneous distracters (thus necessitating “feature search”), whereas in another context they searched for a shape target amongst homogeneous distracters (thus inducing “singleton search”). Crucially, the two tasks were presented in a blocked manner, and presented on contextually distinct backgrounds. For example, the feature search trial block was presented on “forest scene” backgrounds, whereas the singleton search trial block was presented on “city scene” backgrounds. At test, participants were required to search for a shape singleton in the presence of an irrelevant color singleton (identical to that of Theeuwes, 1991, 1992), but the search stimuli were randomly presented on either “forest” or “city” backgrounds from one trial to the next. The key finding was that when search trials were presented on a background that was previously associated with feature search mode, capture effects were not evident, whereas when trials were presented on a background that was previously associated with singleton search mode, robust capture effects were observed. The authors concluded that the task-irrelevant context cued the retrieval of prior experiences associated with that context, and that the attentional set associated with those prior experiences then constrained the influence of singletons on performance. Furthermore, this context-specific effect occurred in the absence of any conscious strategizing or intention on the part of the participants, and thus constitutes evidence of *implicit* control over the capture of attention by an irrelevant singleton.

### The present study

Cosman and Vecera (2012) provided an initial demonstration that context-specific learning processes can modulate the capture of attention by an irrelevant singleton. Our aim here was to use a different tool to offer converging evidence for this idea. In particular, we borrowed from the literature on proportion congruent effects in selective attention to create a method that measures a novel context-specific learning effect on attention capture.

It is well known that Stroop effects are larger when an observer performs in a context in which there is a high proportion of congruent trials (e.g., the word BLUE presented in blue) relative to a context in which there is a low proportion of congruent trials (Logan and Zbrodoff, 1979). More recently, it has been shown that such proportion congruent effects can occur when high and low proportion congruent conditions are associated with distinct items (Jacoby et al., 2003). For example, Jacoby et al. (2003) employed two sets of color words in a Stroop task and manipulated proportion congruency separately for each set, such that one set of words (i.e., “red,” “yellow,” and “white”) were highly likely to appear in their congruent ink color, whereas the other set of words (i.e., “black,” “blue,” and “green”) were highly likely to appear in an incongruent ink color. Despite the fact that these item types were intermixed at random during the experiment, participants demonstrated smaller Stroop effects for the mostly incongruent items relative to the mostly congruent items; an item-specific proportion congruency (ISPC) effect. Similarly, if one associates the probability of congruency with a distinct context such as screen location, one can observe smaller Stroop effects for items that appear in the mostly incongruent location relative to items that appear in a mostly congruent location (Crump et al., 2006; Crump and Milliken, 2009).

Item/context-specific proportion congruent effects appear to be quite robust, as they have now also been reported in a picture-word Stroop task (Bugg et al., 2011), a consecutive trial variant of the Stroop task (Milliken et al., 2012), the flanker task (Lehle and Hübner, 2008), and a global-local letter identification task (Shedden et al., 2013). For a recent review of proportion congruency effects, see Bugg and Crump (2012). In general these results are consistent with the idea that context-sensitive processes can constrain attention to task-irrelevant dimensions in a range of selective attention contexts. Moreover, this process appears to be flexible, leading to trial-to-trial shifts in control over processing, and yet is often unaccompanied by awareness of those shifts by the participant.

We adapted the proportion congruent method to an attention capture method in the present study. Observers searched for a shape singleton target in the presence of an irrelevant color singleton, as in the original experiments conducted by Theeuwes (1991, 1992). However, our experimental design differed in several ways. First, on some trials the color singleton and shape singleton were distinct objects in the display (as in previous work), hereafter referred to as “incongruent” trials, whereas on other trials the color singleton and shape singleton were one and the same object, hereafter referred to as “congruent” trials. Note that on congruent trials, although the color singleton is nominally irrelevant to the task, capture of attention by the color singleton will guide attention to the relevant shape singleton. Second, we manipulated the proportion of trials on which the color and shape singletons were congruent, with congruent trials occurring on either 80 or 20% of trials.

Third, and most importantly, rather than manipulating the proportion congruent between blocks, which would allow different strategies to affect performance for the two proportion congruent conditions, proportion congruent was instead manipulated between items mixed within blocks. Specifically, proportion congruent was linked to the type of shape distractor that occurred on a trial. When one shape (e.g., square) served as the distracter, the trial was highly likely to be congruent, whereas when another shape (e.g., diamond) served as the distracter, the trial was highly likely to be incongruent. Thus, although the proportions of congruent and incongruent trials were equal within each experiment, we were interested in whether participants would learn that when a particular distracter context was present, attention could reliably be guided by the color singleton to the shape singleton. Likewise, when another distracter was present, participants might learn that capture of attention by the color singleton ought to be avoided. If this learning were to occur, then we would expect to observe a larger influence of the irrelevant color singleton on performance in the high-proportion congruent context than in the low-proportion congruent context. Our measure of the influence of the irrelevant color singleton on performance was the difference in performance between congruent and incongruent trials, and thus our aim was to measure an ISPC effect, as per Jacoby et al. (2003).

## Experiment 1

The purpose of Experiment 1 was to assess whether item-specific control over visual selection would manifest in an attention capture paradigm. To do so, we employed a procedure similar to Theeuwes (1992), but with two trial types that differed in terms of the distracter shape (either squares or diamonds). The task of the participant was to locate and respond to the singleton circle in each display. Crucially, there was an irrelevant color singleton on *every* trial. When one type of distracter was present (e.g., squares) the color singleton was likely to be the circle singleton (the high proportion congruent condition), whereas when the other type of distracter was present (e.g., diamonds) the color singleton was *un*likely to be the circle singleton (i.e., the low proportion congruent condition). If item-specific learning (and context-specific expression of that learning) is possible, then observers should learn to allow the capture of attention by the color singleton in the high proportion congruent context, and to prevent such capture in the low proportion congruent context.

### Method

#### Participants

All data collection reported in this article was approved by the McMaster Research Ethics Board. Twenty undergraduate students (14 females) from McMaster University, between the ages of 18 and 21, provided informed consent and received course credit for their participation in this study. All participants reported normal color vision, and normal or corrected-to-normal visual acuity.

#### Apparatus and stimuli

Stimuli were presented on a Sony 15-inch color monitor that was connected to a Comptech Intel Pentium computer. The experiment was run using Micro Experimental Laboratory (MEL2) software (Schneider, 1988). The viewing distance from the computer screen was approximately 57 cm.

The stimulus display consisted of 5, 7, or 9 items equally spaced around the fixation dot (0.2° of radius) on an imaginary circle whose radius was 7.8°. The target stimulus was always an outlined red or green circle (1.0° of radius) that contained a white line segment (0.9° of height and 0.1° of width) that was oriented either horizontally or vertically. The distractor stimuli were red or green outlined squares (1.9° of height and width) or diamonds (2.7° of height and width), each containing a line segment that was tilted randomly 22.5° to either side of the horizontal or vertical plane. In each display, one shape always appeared in a unique color and there was an equal probability that the uniquely colored shape would be the target circle or a distractor. Target and distracter locations were determined randomly on each trial. Finally, each search display contained five, seven, or nine items (one of which was the odd-shape target). There were equal numbers of trials of each display size, and display size was also determined randomly on a trial by trial basis.

#### Procedure

**Figure 1:**
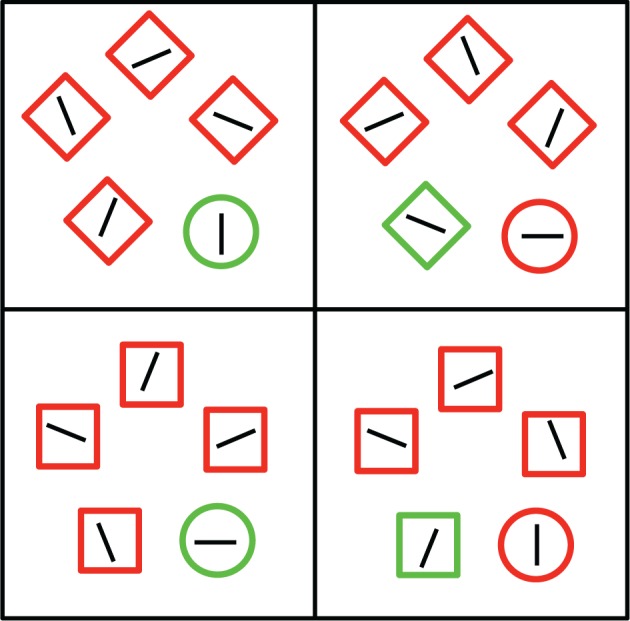
**Examples of the four trial types present in Experiment 1**. Participants were always instructed to search for the unique circle in the display. The left side depicts congruent trials, in which the target shape was also a color singleton. The right side depicts incongruent trials, in which one of the distractor shapes was also a color singleton. Images are not to scale.

Participants were informed that they would be shown an array of shapes and would be required to locate the unique circle in the display. Their task was to respond to the orientation of the line segment within the target circle by pressing the key labeled “H” (which was the “C” key on the keyboard) for a horizontal line segment and by pressing the key labeled “V” (which was the “M” key on the keyboard) for a vertical line segment. Each trial began with a blank screen that lasted for 1000 ms followed by the onset of the search array, which remained on the screen until a response was made. Speed and accuracy were stressed to the participants. The first block of the experiment was a practice block containing 20 trials, while the three remaining experimental blocks contained 240 trials each. For half of the participants (the high-diamond group), the circle target was also the odd-colored item (a congruent trial) on 80% of the trials with diamond distractors, and on 20% of the trials with square distractors. For the other half of the participants (the high-square group), the circle target was congruent for 80% of the trials with square distractors for 20% of the trials with diamond distractors. There were an equal number of trials presented with square and diamond distracters. The two trial types are depicted in Figure 1.

At the end of the test session, participants were asked questions intended to assess their awareness of the proportion congruent manipulation within each trial type. Specifically, they were shown an illustration of a congruent and an incongruent trial for each of the two distracter types. They were then asked to estimate the relative proportions of congruent and incongruent trials for each distractor type, with the constraint that these two estimates, for each distractor type, must add to 1.0. The images of the four trial types were labeled (e.g., “square distracters—congruent trial”) and participants wrote down their proportion estimates next to the corresponding label on a separate sheet of paper. The experimental session lasted approximately 30 min.

### Results

Correct response times (RTs) in each condition were submitted to an outlier elimination procedure that excluded RTs less than 200 ms and greater than 2500 ms, resulting in the removal of 0.6% of trials from further analysis. Mean correct RTs were then computed from the remaining observations, and these mean RTs and corresponding error rates were submitted to a mixed design ANOVA that included congruency (congruent/incongruent), proportion congruent (high/low), display size (5/7/9), and block (1/2/3) as within-subject factors, and group (high-diamond/high-square) as a between-subjects factor. Mean RTs and error percentages in each condition, collapsed across participants and block, are displayed in Table 1.

**Table 1:** **Mean response times and error percentages (in parentheses), collapsed across block, for each condition in Experiment 1**.

**Distractor type**				
	**Square proportion congruent**		**Diamond proportion congruent**	
**Congruency**	**High**	**Low**	**High**	**Low**
**DISPLAY SIZE 5**				
Cong	749 (2.4)	736 (6.4)	703 (4.3)	693 (2.1)
Incong	856 (3.3)	784 (5.2)	801 (3.8)	815 (3.1)
Difference	107 (0.9)	48 (−1.2)	98 (−0.5)	122 (1.0)
**DISPLAY SIZE 7**				
Cong	724 (3.1)	715 (3.8)	700 (4.8)	721 (1.7)
Incong	811 (0.8)	789 (5.4)	798 (3.8)	850 (2.7)
Difference	87 (−2.3)	74 (1.6)	98 (−1.0)	129 (1.0)
**DISPLAY SIZE 9**				
Cong	713 (2.0)	697 (5.4)	695 (4.0)	729 (2.5)
Incong	867 (4.6)	793 (5.5)	852 (2.6)	883 (2.7)
Difference	154 (2.6)	96 (0.1)	157 (−1.4)	154 (0.1)

*Participants searched for an odd target circle amidst either square or diamond distractors (Cong, Congruent; Incong, Incongruent. Difference = Incongruent − Congruent)*.

#### Response times

**Figure 2:**
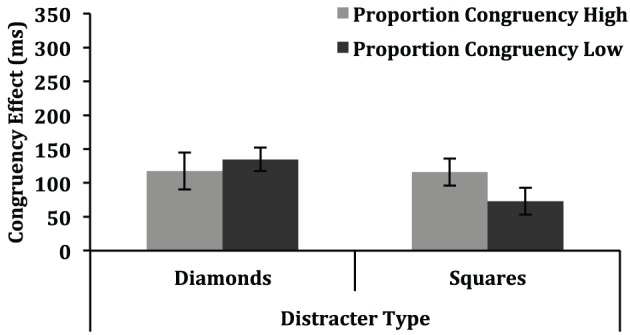
**Congruency effects (mean incongruent RT—mean congruent RT) in Experiment 1 are depicted as a function of proportion congruency (high, low) and distracter type (diamond, square)**. Error bars represent one standard error of the mean.

Our primary aim was to assess whether the nominally irrelevant singleton color contributed to performance more for the high proportion congruent than for the low proportion congruent condition. In this light, the key result was a non-significant interaction between proportion congruent and congruency (*p* = 0.25), indicating that capture by the singleton color did not contribute differentially to performance in the high proportion congruent and low proportion congruent conditions. The mean congruency effects for the high proportion congruent and low proportion congruent conditions, plotted separately for the square distractor and diamond distractor items, are depicted in Figure 2.

There were a number of additional significant effects in the overall ANOVA that were of secondary interest. A full description of these effects can be found in Supplementary materials.

#### Accuracy

An analysis of error percentages that corresponded to that conducted for the RTs revealed no significant main effects, and the key interaction between proportion congruent and congruency was also not significant (*p* = 0.33). Although there were three significant three-way interactions in this analysis, none of them involved both the congruency and proportion congruent factors, and none of them replicated in a following experiment. In addition, the 5-way interaction involving all factors in the analysis was significant, but again it was not replicated in a following experiment.

#### Awareness

Participants' estimates of the proportion of congruent trials for each of the high proportion congruent and low proportion congruent distracter types (provided at the end of the experimental session) are presented in Table 2. These estimates were compared using a matched sample *t*-test and did not differ significantly. This result is consistent with the view that participants were not explicitly aware of the item-specific proportion congruent manipulation.

**Table 2:** **Participants' mean proportion congruency estimates for Experiments 1 and 2**.

		**Trial type**	
	**Proportion congruent**	**Congruent**	**Incongruent**
Experiment 1	High	0.50	0.50
	Low	0.51	0.49
Experiment 2	High	0.51	0.49
	Low	0.45	0.55

### Discussion

The purpose of Experiment 1 was to determine whether learning of the relation between distracter type (context) and congruency between color and shape singletons would aid search for a shape singleton. If so, then low proportion congruent distracters ought to have cued search processes that discouraged color singleton processing, while high proportion congruent distracters ought to have cued search processes that encouraged color singleton processing. In turn, congruency effects ought to have been larger for the high proportion congruent distractor trials than for the low proportion congruent distractor trials. The results were clear. There was no difference in congruency effects between the two contexts; that is, no ISPC effect was observed.

Although the results of this experiment might be taken to imply that context-specific learning cannot influence performance in this attention capture task, there are at least two alternative interpretations. One possibility is that the target shape (a circle) was so distinct from both of the distracter types (squares and diamonds) that bottom-up salience on its own guided a rapid search for the target, leaving little opportunity for context-specific learning to contribute to search for the target. A second possibility is that the high level of perceptual similarity between the two distracter items hindered item-specific learning. Indeed, squares and diamonds share the same perceptual form, and differ only in terms of orientation. We addressed these possibilities in Experiment 2.

## Experiment 2

The purpose of Experiment 2 was to address whether the lack of an ISPC effect in Experiment 1 was due to the perceptual similarity of the two distracter types in that experiment. To that end, observers in the present experiment searched for a unique square in each display amongst homogeneous diamond or circle distracters. We hypothesized that the perceptual distinctiveness of the circle and diamond distracters might allow observers to associate the high and low proportion congruent contingencies with the two distractor types. If this learning occurs, then it may guide search such that performance is influenced more by the irrelevant color singleton in the high proportion congruent context than in the low proportion congruent context. That is, an ISPC effect ought to emerge, with larger congruency effects for the high proportion congruent trials than for the low proportion congruent trials.

An alternative possibility addressed by this experiment is that ISPC effects may be related to the salience of the target relative to the distractors. In Experiment 1, the circle targets were highly distinct from both the square and diamond distractors, producing relatively small congruency effects. In the present Experiment, it was expected that the square targets would be much more salient amidst circle distractors than amidst diamond distractors. If the ISPC effect is related to the difficulty of singleton search, then it might well occur for the difficult searches for a square amidst diamond distractors, but not for the easier searches for a square amidst circle distractors.

### Method

#### Participants

Twenty undergraduate students (19 females) from McMaster University, between the ages of 18 and 21 provided informed consent and participated for course credit. All participants reported normal color vision and normal or corrected-to-normal visual acuity.

#### Stimuli and apparatus

The apparatus and stimuli were identical to that in Experiment 1 with the exception that the target stimulus was now a unique square shape appearing with either all circle distractors or all diamond distractors.

#### Procedure

**Figure 3:**
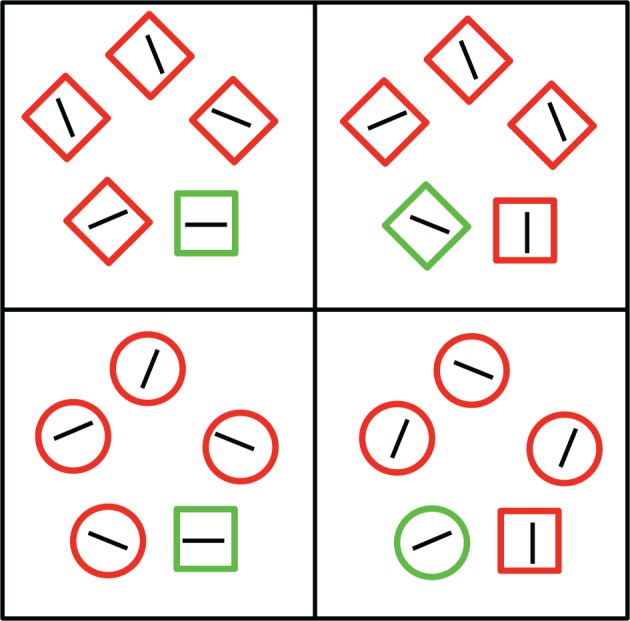
**Examples of the four trial types present in Experiment 2**. Participants were always instructed to search for the unique square in the display. The left side depicts congruent trials, in which the target shape was also a color singleton. The right side depicts incongruent trials, in which one of the distractor shapes was also a color singleton. Images are not to scale.

Experiment 2 followed the same procedure and design as Experiment 1 with the exception that participants were instructed to search for a unique square target. Thus, for half of the participants, the proportion of congruent trials was 0.80 for diamond distractors and 0.20 for circle distractors (the high-diamond group), whereas for the other participants the proportion of congruent trials was 0.80 for circle distractors (the high-circle group) and 0.20 for diamond distractors. Examples of the stimuli are presented in Figure 3. Awareness of the relation between distracter type and proportion congruency was assessed following the experimental session in the same manner as in Experiment 1.

### Results

Correct RTs less than 200 ms or greater than 2500 ms (0.2% of the observations) were again excluded from analyses. Mean correct RTs and error rates for each condition were then computed and submitted to a mixed design ANOVA that included congruency (congruent/incongruent), proportion congruency (high/low), display size (5/7/9), and block (1/2/3) as within-subject factors and group (high-diamond/high-circle) as a between-subjects factor. Mean RTs and error percentages in each condition, collapsed across participants and block, are displayed in Table 3.

**Table 3:** **Mean response times and error percentages (in parentheses), collapsed across block, for each condition in Experiment 2**.

**Distractor type**				
	**Circle proportion congruent**		**Diamond proportion congruent**	
**Congruency**	**High**	**Low**	**High**	**Low**
**DISPLAY SIZE 5**				
Cong	725 (3.9)	761 (2.9)	789 (3.1)	792 (3.8)
Incong	797 (5.8)	811 (2.0)	1028 (3.3)	945 (2.5)
Difference	72 (1.9)	50 (−0.9)	261 (0.2)	153 (−1.3)
**DISPLAY SIZE 7**				
Cong	732 (2.4)	718 (2.5)	785 (2.7)	780 (2.9)
Incong	804 (1.3)	819 (3.0)	1062 (1.3)	1010 (1.8)
Difference	72 (−1.1)	101 (0.5)	277 (−1.4)	230 (−1.1)
**DISPLAY SIZE 9**				
Cong	734 (3.6)	767 (2.1)	791 (2.4)	802 (4.2)
Incong	822 (3.3)	850 (2.8)	1216 (2.5)	1105 (3.3)
Difference	88 (−0.3)	83 (0.7)	425 (0.1)	303 (−0.9)

*Participants searched for an odd target square amidst either circle or diamond distractors (Cong, Congruent; Incong, Incongruent. Difference = Incongruent − Congruent)*.

#### Response times

Again, the key issue was whether the irrelevant singleton color contributed to performance more for the high proportion congruent than for the low proportion congruent condition. In contrast to Experiment 1, the interaction between proportion congruent and congruency was significant, [*F*_(1, 18)_ = 6.36, *p* = 0.021, η^2^_*p*_ = 0.26]. The congruency effect was larger for the high proportion congruent condition (195 ms) than for the low proportion congruent condition (153 ms). This result suggests that the irrelevant singleton color guided search more in the high proportion congruent condition than in the low proportion congruent condition.

**Figure 4:**
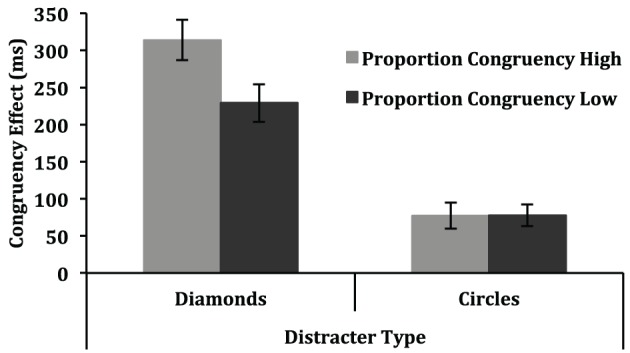
**Congruency effects (mean incongruent RT—mean congruent RT) in Experiment 2 are depicted as a function of proportion congruency (high, low) and distracter type (diamond, circle)**. Error bars represent one standard error of the mean.

The 3-way interaction between group, proportion congruent and congruency was also significant, [*F*_(1, 18)_ = 133.83, *p* < 0.001, η^2^_*p*_ = 0.88]. Note that the interpretation of this interaction is complicated by the fact that the distractor types associated with the high and low proportion congruent conditions changed across groups. As a result, this interaction is sensitive to differences in the magnitude of the congruency effect for the two distractor types. For example, if diamond distractors typically produce a 300 ms congruency effect, and circle distractors typically produce a 100 ms congruency effect, then counterbalancing which of these two distractor types is associated with the high and low proportion congruent condition across groups will affect the proportion congruent by congruency interaction in a spurious manner. Without any influence of proportion congruent at all, the difference in the congruency effect for the two proportion congruent conditions would be 200 ms for one group and −200 ms for the other group. A more relevant issue for the present purpose is whether the ISPC effect (i.e., the difference in congruency effects as a function of proportion congruent) varied across the two distractor types. Such an effect would also contribute to the three-way interaction between group, proportion congruent and congruency, but must be evaluated separately from the spurious influences described above. To address this issue, separate ANOVAs were conducted for the diamond distractor and circle distractor trials, each of which treated proportion congruency as a between-subject variable, and congruency, display size and block as within-subject variables. The proportion congruent effects for the two item types are displayed in Figure 4. In the analysis of the diamond distractor trials, the interaction between proportion congruent and congruency was significant, [*F*_(1, 18)_ = 5.16, *p* = 0.05, η^2^_*p*_ = 0.22], with a larger congruency effect for the high proportion congruent condition (314 ms) than for the low proportion congruent condition (229 ms). In contrast, for the circle distractor trials, the interaction between proportion congruent and congruency was clearly not significant, *F* < 1. In other words, an ISPC effect was observed for the more difficult diamond distractor search trials but not for the easier circle distractor search trials.

There were a number of additional significant effects in the overall ANOVA that were of secondary interest. A full description of these effects can be found in Supplementary materials.

#### Accuracy

The analysis of error percentages revealed a main effect of display size, [*F*_(2, 36)_ = 3.67, *p* = 0.035, η^2^_*p*_ = 0.17], with the highest error rate for display size 5 (3.4%) and the lowest error rates for display size 7 (2.2%). More important, although the interaction between proportion congruent and congruency was not significant (*F* < 1), the pattern of errors was consistent with the interaction described for the RTs. There were two 4-way interactions that reached significance; the interaction between proportion congruent, congruency, display size, and block, [*F*_(4, 72)_ = 2.71, *p* = 0.037, η^2^_*p*_ = 0.13], and the interaction between group, congruency, display size, and block, [*F*_(4, 72)_ = 2.90, *p* = 0.028, η^2^_*p*_ = 0.14]. Examination of the means did not reveal any easily interpretable pattern for these interactions. No other effects in error analysis reached significance.

#### Awareness

Participants' estimates of the proportion of congruent trials for the high proportion congruent and low proportion congruent distracter types are presented in Table 2. These estimates were again compared using a matched sample *t*-test and did not differ significantly, suggesting that participants were unaware of the item-specific proportion congruent manipulation.

### Discussion

In Experiment 2, the perceptual distinctiveness of the two distracter types was increased relative to Experiment 1, with the idea that it might allow observers to better differentiate the two search contexts (i.e., the high and low proportion congruent contexts). Better differentiation of the two search contexts might then allow participants to learn the association between the different levels of proportion congruent that were associated with the two search contexts. If this were to occur, then an ISPC effect might occur in the second experiment where it did not occur in Experiment 1. Indeed, a significant ISPC effect was observed in Experiment 2.

However, the increased perceptual distinctiveness of the two distractor types in this experiment was also accompanied by an asymmetry between the two distractor types in search efficiency; square targets were much easier to locate with circle distractors than with diamond distractors (see Table 3). If more difficult search is accompanied by greater processing of the distractors, then the association between proportion congruent and distractor shape might be learned more effectively for the diamond distractors than for the circle distractors. In turn, if learning of this association underlies the ISPC effect, then an ISPC effect ought to be observed for the diamond distractor trials but not for the circle distractor trials. Indeed, this is precisely what was observed in Experiment 2. We therefore conclude that implicit learning of the utility of attending or ignoring the color singleton *can* occur, but only when search is sufficiently difficult. We looked to extend these findings in Experiment 3.

## Experiment 3

Search difficulty appears to be critical to the implicit learning effect observed in Experiment 2. However, it is unclear what mechanisms associated with difficult but not easy search are responsible for this effect. In the present experiment, we examined the possibility that the more difficult search conditions in Experiment 2 were associated with a qualitatively different search process than the easier search conditions, and that the presence or absence of the ISPC effect may be related to this processing difference. In particular, we focused on a distinction similar to that between singleton search and feature search modes (Bacon and Egeth, 1994).

Although participants in all conditions knew the identity of the target shape in advance of the search display in Experiment 2, we speculated that use of this information about target identity (as in feature search mode) may have varied across the easy and difficult search conditions. For example, when participants searched for a singleton square amidst circle distractors, the high salience of the singleton square might have led to relatively little top-down use of information about target shape identity in the search process. In contrast, when participants searched for a singleton square amidst diamond distractors, the relatively low salience of the singleton square might have led to more top-down use of information about target shape identity in the search process. As such, it is possible that the implicit learning that supports the ISPC effect is related to top-down use of target shape identity, which may have been greater for diamond distractors than for circle distractors.

To test this particular variant of our search difficulty hypothesis, in the present experiment we varied foreknowledge of target shape identity orthogonally with distractor type. The design was similar to that of Experiment 2, with the exception that the target was either a square (as in Experiment 2) or a triangle. In addition, on half of the trials, the participants received an informative cue that indicated, with 100% validity, the target shape on that trial (making these trials functionally identical to those in Experiment 2). On the other half of the trials, participants received an uninformative cue, indicating only that the target shape would be either a triangle or a square. If use of top-down knowledge of target identity is critical to the ISPC effect in Experiment 2, then the ISPC effect might well depend on the cueing condition. In particular, for the more difficult search trials with diamond distractors, an ISPC effect might be expected with informative cues but not with uninformative cues. For the easier search trials with circle distractors, assuming that bottom-up singleton salience predominates, we might expect no ISPC effect for both informative and uninformative cues.

### Method

#### Participants

Forty undergraduate students from McMaster University, between the ages of 18 and 32 provided informed consent and participated for course credit. All participants reported normal color vision and normal or corrected-to-normal visual acuity.

#### Stimuli and apparatus

The apparatus was identical to that in Experiments 1 and 2. The stimulus display was very similar to Experiment 2 except that the target stimulus could either be an outlined square (1.9° of height and width) or an outlined triangle (2.3° of height and base width). Both targets appeared with either all diamond or all circle distractors, and all items were equally likely to appear in red or green. Unlike the previous experiments, however, a centrally-presented cue preceded each stimulus display. The cue was either an uninformative question mark (1.1° of height and 0.5° of width) or an informative target shape (e.g., square or triangle). The informative shape cues indicated the singleton target shape with 100% validity. Both the uninformative and informative cues appeared in white against the black background.

#### Procedure

Participants were informed that they would be shown an array of shapes and would be required to locate the unique shape in the display, which could either be a square or a triangle. They were also told that on cued trials, a cue in the form of a target shape would appear before the stimulus display to indicate whether the upcoming target was a square or a triangle. On uncued trials, however, a question mark would appear before the stimulus display, in which case participants were instructed to simply search for the unique shape (e.g., either a square or a triangle) in the display. As in Experiments 1 and 2, their task was to respond with a keyboard response to the orientation of the line segment within the target.

The first block of the experiment was a practice block containing 20 trials. To compensate for the addition of a second target item in Experiment 3, the number of experimental blocks increased from three blocks of 240 trials in Experiment 2 to six blocks of 240 trials. Each trial began with a cue presented for 750 ms. The search display appeared 500 ms after offset of the cue. The search display contained a central fixation dot in place of the cue and remained in view until the participant's keyboard response. Following the response, there was a 500 ms inter-trial interval during which the screen was blank. Due to the complexity of the design, participants in Experiment 3 were not asked to estimate proportion congruency for each condition after the test session. The experimental session lasted approximately 50 min.

**Figure 5:**
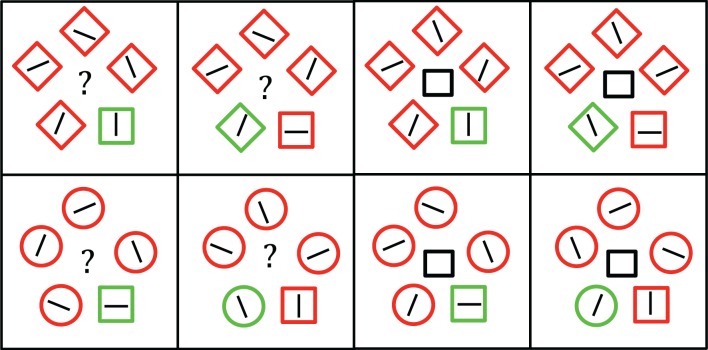
**Examples of the eight trial types present in Experiment 3 when the target was a square (the triangle target condition is not shown)**. The four trial types on the left side are un-cued trials, where the participant was only instructed to search for the unique shape in the display. The four trial types on the right side are cued trials, where the centrally-presented cue indicated which of two shapes would be the upcoming target. In both conditions, half of the trials were congruent and half of the trials were incongruent. In the actual experiment, the central cues preceded the search array. Images are not to scale.

For half of the participants, the proportion of congruent trials was 0.80 for diamond distractors and 0.20 for circle distractors (high-diamond group), whereas for the other half of participants the proportion congruent was 0.80 for circle distractors and 0.20 for diamond distractors (high-circle group). Within both of these groups, there were equal proportions of cued and uncued trials within each of the conditions tested in Experiment 2. The various conditions tested in this experiment are depicted in Figure 5.

### Results

Correct RTs that were less than 200 ms or greater than 2500 ms (1.1% of observations) were again excluded from analyses, Mean correct RTs were computed from the remaining observations, and these mean RTs and error percentages for each condition were submitted to a mixed factor ANOVA that included congruency (congruent/incongruent), proportion congruent (high/low), display size (5/7/9), and cueing (cued/uncued) as within-subject factors, and group (high-diamond/high-circle) as a between-subjects factor. Mean RTs and error percentages in each condition, collapsed across participants, are displayed in Tables 4A,B.

**Table 4A:** **Mean response times and error percentages (in parentheses) for the informative cued trials, collapsed across target (triangle/square) and block, in Experiment 3**.

**Distractor type**				
	**Circle proportion congruent**		**Diamond proportion congruent**	
**Congruency**	**High**	**Low**	**High**	**Low**
**DISPLAY SIZE 5**				
Cong	780 (3.9)	689 (3.5)	749 (2.9)	879 (3.1)
Incong	872 (7.2)	752 (2.4)	985 (4.6)	971 (5.3)
Difference	92 (3.2)	63 (−1.1)	236 (1.7)	92 (1.8)
**DISPLAY SIZE 7**				
Cong	767 (4.4)	703 (2.0)	753 (3.1)	805 (2.5)
Incong	859 (5.0)	756 (3.1)	1058 (3.0)	1051 (6.5)
Difference	92 (0.6)	53 (1.1)	305 (−0.1)	246 (4.0)
**DISPLAY SIZE 9**				
Cong	764 (3.4)	711 (2.5)	750 (2.9)	827 (2.0)
Incong	842 (6.2)	801 (3.6)	1110 (2.1)	1138 (6.5)
Difference	78 (2.8)	90 (1.1)	360 (−0.8)	311 (4.5)

*Participants searched for an odd target square or triangle amidst either circle or diamond distractors (Cong, Congruent; Incong, Incongruent. Difference = Incongruent − Congruent)*.

**Table 4B:** **Mean response times and error percentages (in parentheses) for the uninformative cued (uncued) trials, collapsed across target (triangle/square) and block, in Experiment 3**.

**Distractor type**				
	**Circle proportion congruent**		**Diamond proportion congruent**	
**Congruency**	**High**	**Low**	**High**	**Low**
**DISPLAY SIZE 5**				
Cong	827 (4.3)	722 (4.0)	800 (2.6)	853 (2.5)
Incong	934 (9.8)	801 (3.6)	1061 (5.6)	1059 (8.9)
Difference	107 (5.5)	79 (−1.4)	261 (3.0)	206 (6.4)
**DISPLAY SIZE 7**				
Cong	811 (3.9)	739 (2.5)	773 (3.0)	871 (3.1)
Incong	1002 (8.3)	808 (3.0)	1111 (2.0)	1087 (9.8)
Difference	191 (4.4)	69 (0.5)	338 (−1.0)	216 (6.7)
**DISPLAY SIZE 9**				
Cong	809 (2.8)	747 (6.0)	758 (3.4)	886 (3.5)
Incong	984 (9.2)	861 (3.2)	1176 (3.2)	1142 (10.5)
Difference	175 (6.4)	114 (−2.8)	418 (−0.2)	256 (7.0)

*Participants searched for an odd target square or triangle amidst either circle or diamond distractors (Cong, Congruent; Incong, Incongruent. Difference = Incongruent − Congruent)*.

#### Response times

**Figure 6:**
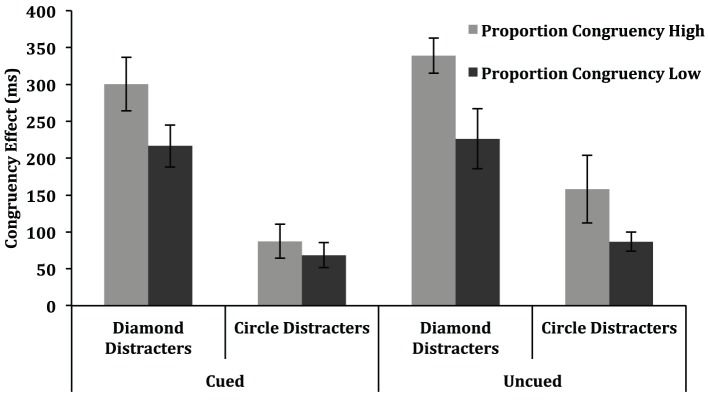
**Congruency effects (mean incongruent RT—mean congruent RT) in Experiment 3 are depicted as a function of proportion congruency (high, low), distracter type (diamond, square), and whether target identity was cued or uncued**. Error bars represent one standard error of the mean.

As in Experiment 2, there was a significant interaction between proportion congruent and congruency, [*F*_(1, 18)_ = 13.88, *p* = 0.002, η^2^_*p*_ = 0.44], with the congruency effect being larger for the high proportion congruent condition (221 ms) than for the low proportion congruent condition (150 ms). Also as in Experiment 2, the three-way interaction between group, proportion congruent and congruency was significant, [*F*_(1, 18)_ = 78.41, *p* < 0.001, η^2^_*p*_ = 0.81]. As noted in Experiment 2, this interaction could be driven entirely by larger congruency effects for diamond distractor trials than for circle distractor trials (see Table 4). To assess whether this interaction also reflects differences in the magnitude of the proportion congruent by congruency interaction for the two distractor shapes, separate ANOVAs were conducted for the diamond distractor and circle distractor trials, each of which treated proportion congruency as a between-subjects variable, and congruency, display size and cueing as within-subject variables. The proportion congruent effects for the two distractor types, plotted separately for cued and uncued trials, are displayed in Figure 6.

In the analysis of the diamond distractor trials, the interaction between proportion congruent and congruency was significant, [*F*_(1, 18)_ = 6.57, *p* = 0.020, η^2^_*p*_ = 0.27], with a larger congruency effect for the high proportion congruent condition (320 ms) than for the low proportion congruent condition (221 ms). In contrast, in the analysis of the circle distractor trials, the interaction between proportion congruent and congruency was not significant, *p* > 0.20. Together, these results offer a close replication of Experiment 2, with the ISPC effect being observed only for the more difficult diamond distractor trials.

Returning to the overall analysis, the key issue to be addressed in this experiment concerned the influence of cueing on the ISPC effect. As such, we next focus on effects involving the cueing factor. The main effect of cueing was significant, [*F*_(1, 18)_ = 10.70, *p* = 0.004, η^2^_*p*_ = 0.37], with faster responses for cued trials (849 ms) than for uncued trials (901 ms). The interaction between cueing and congruency approached significance, [*F*_(1, 18)_ = 4.01, *p* = 0.061, η^2^_*p*_ = 0.18], with the congruency effect being larger for uncued trials (202 ms) than for cued trials (167 ms). These results support the view that participants used the informative cues to facilitate performance. In this light, it is noteworthy that the three-way interaction between proportion congruent, congruency and cueing was not significant, *p* > 15. Examination of the congruency effects in Figure 5 reveals no evidence for the view that the ISPC effect depends on top-down use of the cues. If anything, the trend in these results, though non-significant, is for the ISPC effect to be larger in the more difficult uncued conditions. No other effects involving the cueing factor were significant in the overall analysis.

There were a number of additional significant effects in the overall ANOVA that were of secondary interest. A full description of these effects can be found in Supplementary materials.

#### Accuracy

The analysis of error percentages revealed only a significant interaction between proportion congruent, congruency and display size, [*F*_(2, 36)_ = 4.25, *p* = 0.022, η^2^_*p*_ = 0.19]. Separate analyses of the three display sizes revealed that the proportion congruent by congruency interaction varied across display size in a way that was difficult to interpret, being consistent with the RT pattern for display size 5 (*p* = 0.110), and opposite the RT pattern for display sizes 7 (*p* = 0.025) and 9 (*p* = 0.664). Collapsed across the three display sizes, the proportion congruent by congruency interaction was clearly not significant (*F* < 1), and therefore the pattern of RTs of most interest, the ISPC effect, does not appear to be the result of a speed-accuracy trade-off.

### Discussion

In Experiment 2, an ISPC effect was observed when observers searched for a square target amidst diamond distracters, but not amidst circle distracters. In Experiment 3, we examined whether the ISPC effect occurred in the more difficult search condition in Experiment 2 because participants made greater use of top-down knowledge of target shape to find the target in the difficult search condition (i.e., diamond distractors) than in the easy search condition (i.e., circle distractors). To test this idea, on some trials we presented an informative cue that identified the target shape, while on other trials we presented an uninformative cue that failed to identify the target shape. If use of top-down knowledge of the target shape is critical to the implicit learning that produces the ISPC effect, then the ISPC effect ought to be more pronounced for the informative cue condition than for the uninformative cue condition. The results were clear. Although an ISPC effect was again observed for the difficult diamond distracter condition, and not for the easier circle distractor condition, this effect did not differ as a function of whether the cue was informative or not. In fact, in terms of magnitude, the ISPC effect was actually larger for the uninformative cue condition. Furthermore, the observed main effect of cueing indicates that while there was a cost associated with not knowing the target identity in advance, specifically in the difficult search condition, this did not eliminate the ISPC effect for difficult search trials. We conclude that the dependence of the ISPC effect on search difficulty in Experiments 2 and 3 is not related to greater use of top-down knowledge of target shape for difficult searches. We discuss the implications of these findings further in the General Discussion.

## General discussion

The goal of the present series of experiments was to explore whether the processing of an irrelevant color singleton in a task typically used to measure attention capture is subject to implicit learning. To that end, we employed an ISPC manipulation (Jacoby et al., 2003). Specifically, for one set of distracters, the shape singleton (the target item) and the color singleton (the salient distracter) were likely to coincide (i.e., congruent trials). For another set of distracters, the shape singleton and color singleton were likely not to coincide (i.e., incongruent trials). In this way, there was an opportunity for observers to learn that in the high-proportion congruent context, performance would benefit from allowing attention to be captured by the salient color singleton, as it was also the shape singleton target on most of the trials. Similarly, in the low-proportion congruent context, there was an opportunity to learn that performance would benefit from ignoring the salient color singleton, as it was rarely the shape singleton target. We hypothesized that if indeed observers were able to learn the relation between distracter type (context) and the utility of allowing attention to be captured by the color singleton, then congruency effects (the difference in response time between congruent and incongruent trials) would be larger for high proportion congruent items than for low proportion congruent items; an ISPC effect.

Indeed, we observed an ISPC effect, but only when the target singleton and the distracter shapes were perceptually similar, indicating that item-specific learning and/or the expression of that learning only occurs when search is sufficiently difficult. Moreover, the ISPC effect observed here appears not to hinge on top-down use of knowledge about the target shape, as the ISPC effect was observed in Experiment 3 even without a cue indicating the target. Finally, we suggest that the ISPC effect observed here hinges on the *implicit* recruitment of prior learning, rather than on conscious strategy shifts, as observers exhibited no knowledge of the item-specific manipulations on the post-experiment questionnaire administered following Experiments 1 and 2.

The results of the present work are important in several ways: (1) while there are now numerous demonstrations that attention capture effects are modulable by explicit, top-down knowledge and strategy, only one other study (to our knowledge) has demonstrated that such effects are sensitive to *implicit* context-specific knowledge (Cosman and Vecera, 2012); (2) Behavioral effects in several other performance domains have been shown to be modulated by the match or mis-match in task irrelevant contextual information between prior experience and current perception (i.e., proportion congruency effects in the Stroop task—Crump et al., 2006; negative priming effects—Neill, 1997; conflict adaptation effects—Spapé and Hommel, 2008; long-lasting inhibition of return—Wilson et al., 2006; and long-lasting priming effects in singleton search—Thomson and Milliken, 2012, 2013a,b). The present work however, represents the first evidence that properties of the task-relevant stimuli themselves can bias search behavior in the context of the attention capture paradigm; and (3) While most prior studies showing that prior experience can bias the capture of attention by an irrelevant singleton can be explained via automatic, trial-to-trial influences (i.e., the operation of “short-term” priming), the ISPC effect observed here demonstrates that trial history beyond the most recent experience can affect the capture of attention by the most salient item in the search display.

A key finding of the present work is that the ISPC effect seems only to occur when search is perceptually “difficult.” Specifically, when the target shape singleton was too distinct with respect to the distracters (i.e., a square or triangle amongst circles), no evidence of context-specific learning was observed (in both Experiments 2 and 3). The interaction between task difficulty and the ISPC effect was not predicted a priori and deserves further study. Ultimately, how one explains the effect of task difficulty will hinge on interpretation of the ISPC effect in the first place. Under an episodic retrieval view for example, it has long been argued that the implicit effects of prior experience on behavior will only manifest to the extent that the retrieval of prior episodes is faster, or more efficient, than generating the appropriate behavior “from scratch” (or what Logan, 1988, referred to as “algorithmic processing”). To the extent that computing the solution to a problem is more difficult, retrieval of the appropriate solution from memory (given sufficient prior experience with the problem) becomes the more efficient processing route. In the context of the present work, if the time to recruit appropriate similar prior search experiences is longer than the time to locate the target shape in a “bottom-up” (i.e., algorithmic) manner, then no expression of prior knowledge will be observed. It is reasonable to conclude therefore, that when the target shape and the distracter shapes are sufficiently distinct, bottom-up salience guides attention to the target in a more efficient manner than does the retrieval of prior action.

In contrast, one might interpret the ISPC effects seen in the present work not as reflecting the implicit recruitment of episodic representations, but instead perhaps reflecting some form of expectation-based suppression of irrelevant information. For example, the presence of a particular distracter set might lead one to “expect” color to be irrelevant, leading to suppression of low-level representations of color (so-called “dimension-weighting,” see Tollner et al., 2010). Indeed, it has been shown that such suppression at the neural level can unfold in accord with the expectations of the observer in a dynamic trial-to-trial manner (Summerfield et al., 2008). In order for this to occur however, the contingencies between distracter identity and the probability of congruency must be learned in the first place, and such learning likely hinges on the extent to which the distracters are actively attended. To the extent that the perceptual salience of the shape target is especially high with respect to the distracter shapes, bottom-up guidance of attention to the target may preclude learning of the congruency-distracter relation. As a result, when the perceptual discriminability of the target relative to the distracters is too high, distracter identity will not serve as a sufficient cue for expectation-based modulation of task-relevant and irrelevant feature dimensions.

Regardless of whether the ISPC effects observed here derive from the context-specific retrieval of episodic memory representations, or contingency-based modulations of feature dimensions, a worthwhile goal of future work will be to systematically vary search difficulty while implementing a proportion congruency manipulation in the context of the attention capture paradigm. Based on the present work, one would predict a negative relation between the perceptual discriminability of target relative to distracters, and the magnitude of the ISPC effect.

Finally, it should be noted that although the present results show that context-specific guidance of attention to a salient singleton can unfold in a dynamic, implicit, trial-to-trial manner, the locus of this effect in the search process remains unknown. Specifically, the effects of item-specific context may operate to speed attentional allocation the target object on congruent trials, or alternatively, context may serve to increase the speed of attentional disengagement from the distracter object on incongruent trials (or indeed, context may operate on both processes). To address this issue, a baseline condition might be employed in future work, such as a “no color singleton” condition. If RTs were found to be similar in this control condition to those of congruent trials in the present work then the disengagement hypothesis would be favored, however, if RTs in the control condition were slower than on congruent trials in the present work, then the allocation hypothesis might be favored. In addition, eye-tracking measures might be implemented to shed light on this issue. For the moment however, the specific search process affected by context-specific learning in the present procedure remains an open issue.

### Conflict of interest statement

The authors declare that the research was conducted in the absence of any commercial or financial relationships that could be construed as a potential conflict of interest.

## References

[B1] Ásgeirsson Á. G. Kristjánsson Á. (2011). Episodic retrieval and feature facilitation in intertrial priming of visual search. Attent. Percept. Psychophys. 73, 1350–1360 10.3758/s13414-011-0119-5 21491164

[B2] Awh E. Belopolsky A. V. Theeuwes J. (2012). Top-down versus bottom-up attentional control: a failed theoretical dichotomy. Trends Cogn. Sci. 16, 437–443 10.1016/j.tics.2012.06.010 22795563 PMC3426354

[B3] Bacon W. F. Egeth H. E. (1994). Overriding stimulus-driven attentional capture. Percept. Psychophys. 55, 485–496 10.3758/BF03205306 8008550

[B4] Bugg J. M. Crump M. J. (2012). In support of a distinction between voluntary and stimulus-driven control: a review of the literature on proportion congruent effects. Front. Psychol. 3:367 10.3389/fpsyg.2012.00367 23060836 PMC3459019

[B5] Bugg J. M. Jacoby L. L. Chanani S. (2011). Why it is too early to lose control in accounts of item-specific proportion congruency effects. J. Exp. Psychol. Hum. Percept. Perform. 37, 844–859 10.1037/a0019957 20718569

[B6] Burra N. Kerzel D. (2013). Attentional capture during visual search is attenuated by target predictability: evidence from the N2pc, Pd, and topographic segmentation. Psychophysiology 50, 422–430 10.1111/psyp.12019 23418888

[B7] Cosman J. D. Vecera S. P. (2012). Context-dependent control over attentional capture. J. Exp. Psychol. Hum. Percept. Perform. 39, 836–848 10.1037/a0030027 23025581 PMC3924559

[B8] Crump M. J. Gong Z. Milliken B. (2006). The context-specific proportion congruent Stroop effect: location as a contextual cue. Psychono. Bull. Rev. 13, 316–321 10.3758/BF03193850 16893001

[B9] Crump M. J. Milliken B. (2009). The flexibility of context-specific control: evidence for context-driven generalization of item-specific control settings. Q. J. Exp. Psychol. 62, 1523–1532 10.1080/17470210902752096 19370487

[B10] Geyer T. Müller H. J. Krummenacher J. (2008). Expectancies modulate attentional capture by salient color singletons. Vision Res. 48, 1315–1326 10.1016/j.visres.2008.02.006 18407311

[B11] Geyer T. Müller H. J. (2009). Distinct, but top-down modulable color and positional priming mechanisms in visual pop-out search. Psychol. Res. 73, 167–176 10.1007/s00426-008-0207-x 19082623

[B12] Hillstrom A. P. (2000). Repetition effects in visual search. Percept. Psychophys. 62, 800–817 10.3758/BF03206924 10883586

[B13] Hommel B. (1998). Event files: evidence for automatic integration of stimulus-response episodes. Visual Cogn. 5, 183–216 10.1080/713756773

[B14] Hommel B. (2004). Event files: feature binding in and across perception and action. Trends Cogn. Sci. 8, 494–500 10.1016/j.tics.2004.08.007 15491903

[B15] Huang L. Holcombe A. O. Pashler H. (2004). Repetition priming in visual search: episodic retrieval, not feature priming. Mem. Cogn. 32, 12–20 10.3758/BF03195816 15078040

[B16] Jacoby L. L. Lindsay D. S. Hessels S. (2003). Item-specific control of automatic processes: stroop process dissociations. Psychon. Bull. Rev. 10, 638–644 10.3758/BF03196526 14620358

[B17] Lamy D. Yashar A. Ruderman L. (2010). A dual-stage account of inter-trial priming effects. Vision Res. 50, 1396–1401 10.1016/j.visres.2010.01.008 20079758

[B18] Lamy D. F. Kristjánsson Á. (2013). Is goal-directed attentional guidance just intertrial priming? A review. J. Vis. 13, 1–19 10.1167/13.3.14 23818660

[B19] Leber A. B. Egeth H. E. (2006). It's under control: top-down search strategies can override attentional capture. Psychon. Bull. Rev. 13, 132–138 10.3758/BF03193824 16724780

[B20] Lee H. Mozer M. C. Vecera S. P. (2009). Mechanisms of priming of pop-out: stored representations or feature-gain modulations? Attent. Percept. Psychophys. 71, 1059–1071 10.3758/APP.71.5.1059 19525537

[B21] Lehle C. Hübner R. (2008). On-the-fly adaptation of selectivity in the flanker task. Psychon. Bull. Rev. 15, 814–818 10.3758/PBR.15.4.814 18792509

[B22] Logan G. D. (1988). Toward an instance theory of automatization. Psychol. Rev. 95, 492–527 10.1037/0033-295X.95.4.49216099354

[B23] Logan G. D. (1990). Repetition priming and automaticity: common underlying mechanisms? Cogn. Psychol. 22, 1–35 10.1016/0010-0285(90)90002-L

[B24] Logan G. D. Zbrodoff N. J. (1979). When it helps to be misled: facilitative effects of increasing the frequency of conflicting stimuli in a Stroop-like task. Mem. Cogn. 7, 166–174 10.3758/BF03197535

[B25] Maljkovic V. Nakayama K. (1994). Priming of pop-out: I. Role of features. Mem. Cogn. 22, 657–672 10.3758/BF03209251 7808275

[B26] Milliken B. Thomson D. R. Bleile K. MacLellan E. Giammarco M. (2012). Context-specific control and the Stroop negative priming effect. Q. J. Exp. Psychol. 65, 1430–1448 10.1080/17470218.2012.656851 22502818

[B27] Neill W. T. (1997). Episodic retrieval in negative priming and repetition priming. J. Exp. Psychol. Learn. Mem. Cogn. 23, 1291–1305 10.1037/0278-7393.23.6.1291

[B27a] Schneider W. (1988). Micro experimental laboratory: an integrated system for IBM PC compatibles. Behav. Res. Methods Instrum. Comput. 20, 206–217 10.3758/BF03203833

[B28] Shedden J. Milliken B. Watter S. Monteiro S. (2013). Event-related potentials as brain correlates of item specific proportion congruent effects. Conscious. Cogn. 22, 1442–1455 10.1016/j.concog.2013.10.002 24177235

[B29] Spapé M. M. Hommel B. (2008). He said, she said: episodic retrieval induces conflict adaptation in an auditory Stroop task. Psychon. Bull. Rev. 15, 1117–1121 10.3758/PBR.15.6.1117 19001577

[B30] Summerfield C. Trittschuh E. T. Monti J. M. Mesulam M. M. Egner T. (2008). Neural repetition suppression reflects fulfilled perceptual expectations. Nat. Neurosci. 11, 1004–1006 10.1038/nn.2163 19160497 PMC2747248

[B31] Theeuwes J. (1991). Cross-dimensional perceptual selectivity. Percept. Psychophys. 50, 184–193 10.3758/BF03212219 1945740

[B32] Theeuwes J. (1992). Perceptual selectivity for color and form. Percept. Psychophys. 51, 599–606 10.3758/BF03211656 1620571

[B33] Theeuwes J. (2004). Top-down search strategies cannot override attentional capture. Psychon. Bull. Rev. 11, 65–70 10.3758/BF03206462 15116988

[B34] Theeuwes J. Reimann B. Mortier K. (2006). Visual search for featural singletons: no top-down modulation, only bottom-up priming. Visual Cogn. 14, 466–489 10.1080/13506280500195110

[B35] Theeuwes J. Van der Burg E. (2011). On the limits of top-down control of visual selection. Attent. Percept. Psychophys. 73, 2092–2103 10.3758/s13414-011-0176-9 21744177 PMC3204004

[B36] Thomson D. R. D'Ascenzo M. Milliken B. (2013). Learning what to expect: context-specific control over intertrial priming effects in singleton search. Mem. Cogn. 41, 533–546 10.3758/s13421-012-0278-1 23263877

[B37] Thomson D. R. Milliken B. (2011). A switch in task affects priming of pop-out: evidence for the role of episodes. Attent. Percept. Psychophys. 73, 318–333 10.3758/s13414-010-0046-x 21264719

[B38] Thomson D. R. Milliken B. (2012). Perceptual distinctiveness produces long-lasting priming of pop-out. Psychon. Bull. Rev. 19, 170–176 10.3758/s13423-011-0199-1 22231724

[B39] Thomson D. R. Milliken B. (2013a). Contextual distinctiveness produces long-lasting priming of pop-out. J. Exp. Psychol. Hum. Percept. Perform. 39, 202–215 10.1037/a0028069 22506787

[B40] Thomson D. R. Milliken B. (2013b). Revisiting the time course of inter-trial feature priming in singleton search. Psychol. Res. 77, 637–650 10.1007/s00426-012-0455-7 23001245

[B41] Tollner T. Zehetleitner M. Gramann K. Muller H. J. (2010). Top-down weighting of visual dimensions: behavioral and electrophysiological evidence. Vis. Res. 50, 1372–1381 10.1016/j.visres.2009.11.009 19925821

[B42] Treisman A. M. Gelade G. (1980). A feature-integration theory of attention. Cogn. Psychol. 12, 97–136 10.1016/0010-0285(80)90005-5 7351125

[B43] Turatto M. Galfano G. (2001). Attentional capture by color without any relevant attentional set. Percept. Psychophys. 63, 286–297 10.3758/BF03194469 11281103

[B44] Wilson D. E. Castel A. D. Pratt J. (2006). Long-term inhibition of return for spatial locations: evidence for a memory retrieval account. Q. J. Exp. Psychol. 59, 2135–2147 10.1080/17470210500481569 17095492

[B45] Zehetleitner M. Goschy H. Müller H. J. (2012). Top-down control of attention: it's gradual, practice-dependent, and hierarchically organized. J. Exp. Psychol. Hum. Percept. Perform. 38, 941–957 10.1037/a0027629 22506778

