# Supplementary material for: Implicit learning modulates attention capture: evidence from an item-specific proportion congruency manipulation
Source: Front Psychol. 2014 Jun 4;5:551. doi: 10.3389/fpsyg.2014.00551 (PMC4044972; doi:10.3389/fpsyg.2014.00551)
Supplement: Supplementary file 1 [file DataSheet1.DOCX]

**APPENDIX**

**Appendix A – Supplementary description of RT Analyses**

The analysis of RTs in each of Experiments 1, 2, and 3 revealed several significant results above and beyond those described in the main body of the paper. As these results were of less obvious theoretical relevance, they are described in detail here, rather than in the body of the paper, for the benefit of the reader.

**Experiment 1***.* As expected, there was a significant main effect of congruency, *F*(1, 18) = 60.74, *p* < .001, η_p_^2^  = .77, with faster RTs for congruent trials (715 ms) than incongruent trials (825 ms). There was also a significant main effect of block, *F*(2, 36) = 34.21, *p* < .001, η_p_^2^  = .66, with RT decreasing steadily from 841 ms in the first block to 707 ms in the final block. The main effect of display size only approached significance, *F*(2, 36) = 2.63, *p* = .086, η_p_^2^  = .13, indicating at best a modest effect of display size on RT (767 ms, 763 ms, 779 ms for display sizes 5, 7, and 9, respectively).

The analysis also revealed significant two-way interactions between congruency and display size, *F*(2, 36) = 6.71, *p* = .003, η_p_^2^  = .27, and congruency and block, *F*(2, 36) = 7.50, *p* = .002, η_p_^2^  = .29. The congruency effect was largest for display size 9 (140 ms) and smaller for display sizes 5 (96 ms) and 7 (97 ms), and decreased steadily across blocks (144 ms, 110 ms, and 80 ms for blocks 1 to 3).

There were also two significant 3-way interactions, but a close look at the results reveals that they were simple by-products of item differences (diamond distractor items vs square distractor items) in the effects of congruency and display size. For example, the interaction between group, proportion congruent, and congruency was significant,

*F*(1,18) = 8.61, *p* = .009, η_p_^2^  = .32. In fact, this interaction term is sensitive both to differences in the magnitude of the congruency effect for the two distractor types, and to differences in the magnitude of the proportion congruent by congruency interaction for the two distractor types. To appreciate the first of these two influences, consider that when diamond distractor trials switch from being the high proportion congruency trials for one group to low proportion congruency trials for the other group, the item differences in the congruency effect on their own reverse the proportion congruent by congruency interaction. Thus, the larger congruency effect observed for diamond distractor (126 ms) than for square distractor (95 ms) trials (see Table 1) on its own contributes to the significant interaction noted above. To evaluate whether differences in the proportion congruent by congruency interaction for the two items types also contributed to the 3-way interaction, separate ANOVAs were conducted for the diamond distractor and square distractor trials, each of which treated proportion congruency as a between-subject variable, and congruency, display size and block as within-subject variables. The key result from these analyses was that the interaction between proportion congruent and congruency was not significant in either analysis, *p* > .10. As such, the 3-way interaction between group, proportion congruent, and congruency appears to be a spurious consequence of item differences in the congruency effect alone. A similar argument can be made for the significant interaction between group, proportion congruent and display size, *F*(2.36) = 7.58, *p* = .002, η_p_^2^  = .30. In this case, the 3-way interaction appears to be a spurious consequence of a larger effect of display size for the diamond distractor items than for the square distractor items.

**Experiment 2***.* There was a significant main effect of congruency, *F*(1, 18) = 180.66, *p* < .001, η_p_^2^  = .90, with faster RTs for congruent trials (765 ms) than incongruent trials (939 ms). There was also a significant main effect of block, *F*(2, 36) = 57.47, *p* < .001, η_p_^2^  = .76, with RT decreasing monotonically from the first to the third block (950 ms, 829 ms, 777 ms) . The main effect of display size was also significant, *F*(2, 36) = 34.51, *p* < .001, η_p_^2^  = .66, with RT increasing monotonically across the three display sizes (831 ms, 839 ms, 886 ms for display sizes 5, 7, and 9, respectively).

As in Experiment 1, the analysis revealed significant two-way interactions between congruency and display size, *F*(2, 36) = 19.45, *p* < .001, η_p_^2^  = .52, and congruency and block, *F*(2, 36) = 10.46, *p* < .001, η_p_^2^  = .37. The congruency effect increased systematically with increases in display size (128 ms, 170 ms, and 225 ms for displays sizes 5, 7, and 9, respectively), and decreased steadily across blocks (216 ms, 171 ms, and 137 ms for blocks 1 to 3).

There were four additional significant interactions, but in all cases they appeared to have been driven by item differences (diamond distractor vs circle distractor items; see Table 3) that were not of direct relevance to our aims. For example, the interaction between group and proportion congruent was significant, *F*(1, 18) = 221.01, *p* < .001, η_p_^2^  = .92, simply reflecting the slower responses for diamond distractor trials than for circle distractor trials (which switch proportion congruent status across the two groups; see Table 3). There was also a significant interaction between group, proportion congruent and display size, *F*(2, 36) = 9.00, *p* < .001, η_p_^2^  = .33, reflecting a larger effect of display size for the more difficult diamond distractor trials, and a significant interaction between group, proportion congruent and block, *F*(2, 36) = 7.15, *p* = .002, η_p_^2^  = .28, reflecting a larger effect of block for the more difficult diamond distractor trials. Finally, there was a significant 4-way interaction between group, proportion congruent, congruency, and display size, *F*(2, 36) = 3.96, *p* = .028, η_p_^2^  = .18, in this case reflecting a larger congruency by display size interaction (see above) for the more difficult diamond distractor trials.

**Experiment 3.** There was a significant main effect of congruency, *F*(1, 18) = 143.14, *p* < .001, η_p_^2^  = .89, with faster RTs for congruent trials (782 ms) than for incongruent trials (968 ms). The main effect of display size was significant, *F*(2, 36) = 13.98, *p* < .001, η_p_^2^  = .44, with RT increasing monotonically with increases in display size (858 ms, 872 ms, 894 ms for display sizes 5, 7, and 9, respectively). The interaction between congruency and display size was also significant, *F*(2, 36) = 15.66, *p* < .001, η_p_^2^  = .47, with the congruency effect increasing across the three display sizes (142 ms, 189 ms, and 225 ms for displays sizes 5, 7, and 9, respectively. Two additional significant effects owe to the fact that the diamond distractor trials are more difficult than the circle distractor trials, and these two distractor types switch proportion congruent status across groups. This resulted in a significant interaction between group and proportion congruent, *F*(1, 18) = 138.87, *p* < .001, η_p_^2^  = .88, and also a significant 4-way interaction between group, proportion congruent, congruency and display size, *F*(2, 36) = 7.94, *p* = .001, η_p_^2^  = .31. This latter interaction owes to the congruency by display size interaction (see above) being larger for the more difficult diamond distractor trials.
